# Exploring the functional quality attributes of smart home for older adults based on qualitative research and Kano model

**DOI:** 10.3389/fpubh.2025.1541571

**Published:** 2025-07-08

**Authors:** Qin Yang, Peishan Li, Xing Liu, Chunnan Wei

**Affiliations:** ^1^Business School, Sichuan Normal University, Chengdu, China; ^2^School of Economics and Management, Sichuan Technology and Business University, Chengdu, China; ^3^Business School, Sichuan University, Chengdu, China

**Keywords:** ageing, smart home, quality attribute, qualitative research, Kano model

## Abstract

**Background:**

This study investigates the functional attributes of smart home for older adults across different age groups, aiming to identify features that fulfill users’ needs and give convenience, thereby offering scientific guidance for future smart home designs for older adults.

**Methods:**

Employing a multi-stage approach, this study commences with semi-structured interviews with older participants in China, analyzing transcripts using NVivo to guide questionnaire design. Subsequently, a questionnaire survey is administered to older adults in China, with the data processed using the Kano model incorporating the Better-Worse index with sensitivity coefficients.

**Results:**

The findings distinctly demonstrate divergent preferences among different age groups. Specifically, for older adults aged 60–69, health, life and entertainment functions emerge as top priorities, identifying two indicators classified as Must-be quality, five as One-dimensional quality, and one as Attractive quality. In contrast, among those older adults aged 70 and above, emphasis lies predominantly on health, life and emotion functions, identifying one indicator categorized as Must-be quality, six as One-dimensional quality and two as Attractive quality.

**Conclusion:**

This study highlights the existence of significant variations in the needs of different older adult age groups. Through the classification of functional attributes of smart home for older adults, development strategies can be precisely formulated to better meet the needs of different age groups.

## 1 Introduction

According to “World Social Report 2023,” the number of people aged 65 years or older worldwide is projected to more than double, rising from 761 million in 2021 to 1.6 billion in 2050 (1). International communities appeal to catalyze collaborative efforts to improve the well-being of older adults within their homes and communities, fostering their longer and healthier lives (2, 3). “Smart home” is defined as a residence equipped with smart technologies that allow monitoring of its inhabitants and/or encourages independence and the maintenance of good health (4–6). It plays an important role in providing tailored services for older adults to improve their life quality (7–9). Thereby, the concept of “Smart Home for Older Adults” has emerged, which refers to smart home products that provide a more comfortable and healthier lifestyle for older adults, improving their independence and quality of life (10, 11). With the updating of technological products, enhancing the innovative design and development of smart home for older adults holds great significance.

In recent years, research on smart home for older adults has significantly increased spurred by their considerably improved affordability and simplicity (12). However, existing scholarly discourse predominantly gravitates towards technical domains (13), neglecting the perspectives of older users, which is pivotal for fostering smart home implementation and uptake (5). Within the limited corpus of literature that engages with the older adults’ perspective, emphasis has primarily been placed on the physiological dimensions of older adults (8), such as healthcare (14–16), security and monitoring (17), fall detection (18). The exploration of real needs beyond the physiological dimension has been insufficient, impeding the ability to effectively address the intrinsic needs of older adults.

Given that older adults possess different needs (12), influenced by different ages (19, 20), preferences (21), it is essential to classify the attributes of smart home for older adults while taking these factors into account (10). In light of the abovementioned actual issues and research gaps, it emerges as a pressing and paramount concern to explore the functional attributes of smart home for older adults from the perspective of older adults themselves while considering their varying ages and preferences.

Qualitative data can yield meaningful findings that offer unique insights, often unattainable through quantitative research. High-quality qualitative research, when properly managed and utilized, provides deep and nuanced understandings that are crucial in many research contexts (22, 23). Some scholars employed the method of coding interview transcripts, enabling the thematic analysis of qualitative data to obtain core categories and unearth valuable research insights. For instance, Chen et al. (24) used NVivo to code and thematically analyze the interview transcripts to understand mental health issues among Irish employees arising from COVID-19 adaptation. Mazigo et al. (25) used NVivo to digitally record, transcribe, and analyze to get information such as what healthcare workers know about FGS. To improve the quality of qualitative research, we plan to conduct semi-structured interviews, code the qualitative data using NVivo, and perform a thematic analysis to capture core categories. This approach is feasible for preliminarily investigating the market state of smart home for older adults and will guide the questionnaire design.

The Kano model is a simple and effective method for identifying quality attributes, which assesses the attribute directly affecting user satisfaction and their role in quality perception (26, 27). The different Kano models have been used to scientifically and quantitatively explore the specific needs of older adults (28, 29), which helps improve their life quality in later years and decrease physical and psychological stress. Yuan et al. (30) analyzed the needs of telenursing among empty-nest older adults with chronic diseases, using the Better-Worse based Kano model to obtain quality attributes for functions. Similarly, Yuan et al. (31) explored the needs of 285 telenursing for community-dwelling empty-nest older adults through the traditional Kano model and Better-Worse based Kano model. Qiao and Zhang (32) explored the quality needs of landscape architecture courses on MOOC using the traditional Kano model and Better-Worse based Kano model. Given the advantages of the Kano model in the extraction and analysis of commodity attributes, we employ a Kano model incorporating the Better-Worse index with sensitivity coefficients to explore and analyze the specific needs of older adults in different ages for smart home for older adults features.

With the escalating challenge of population aging, older adults emerge as a pivotal demographic for smart homes, necessitating a comprehensive exploration of their distinctive requirements. Therefore, we focus on the actual needs of older users in their later years and approach the study from their perspective. Based on the above-mentioned methods, we initially obtain interview transcripts from older adults through qualitative research and utilize software coding to draw conclusions. These findings subsequently guide the questionnaire design, and in particular assist in the identification of key functions of smart home for older adults. After collecting and processing questionnaires, we analyze the data using the traditional Kano model and the Better-Worse based Kano model, respectively, to explore the attributes of smart home for older adults.

In summary, the main contributions of our research are as follows:

1. Unique perspective: Departing from the vantage point of older users, our study zeroes in on the genuine requirements of the older adults, effectively shifting the focus of smart home technology research toward the needs of older smart home users.
2. Subject categorization: By segmenting the research subjects into distinct age groups and delineating their specific requirements, this paper enables the derivation of more targeted conclusions.
3. Comprehensive research scope: Beyond addressing the physiological needs of older adults, our study employs NVivo software to conduct a qualitative analysis of manuscripts, thereby facilitating questionnaire design and uncovering the authentic needs of older adults in aspects of daily life, emotional well-being, and entertainment.

## 2 Materials and methods

**Figure 1:**
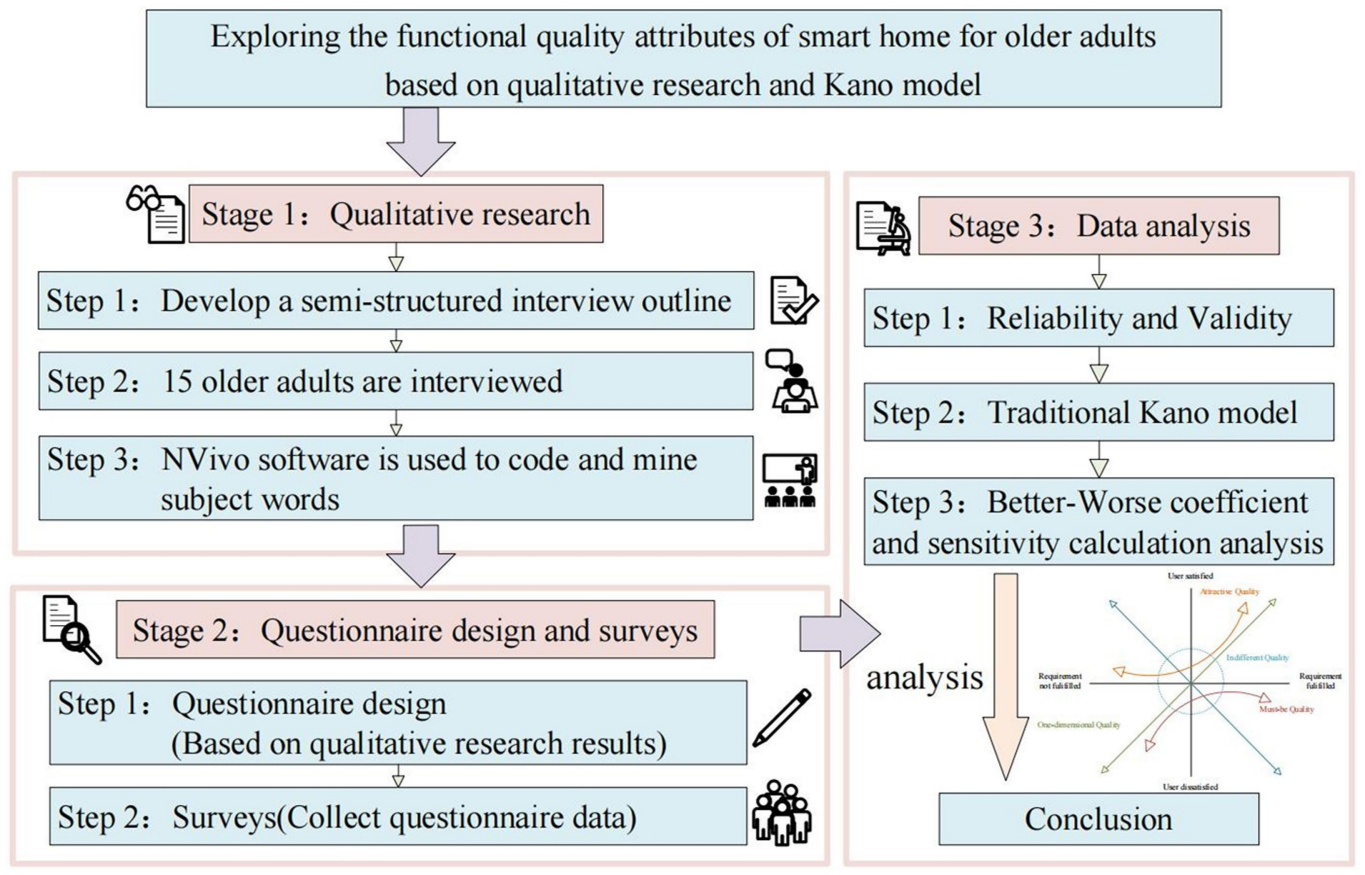
Research process of exploring functional quality attributes of smart home for older adults.

We focus on the functional needs of smart home for older adults and the quality classification of each function. Utilizing a qualitative research approach involving semi-structured interviews, we formulate questionnaires informed by analyzed interview results, followed by an exploration of smart home for older adults function quality attributes using the Kano model. This study is structured into three key stages as shown in Figure 1.

Stage 1: Qualitative research. Initially, 15 older participants are recruited to partake in semi-structured interviews, and all transcripts are subsequently coded using NVivo. Themes extracted from responses are used to identify variables in the questionnaire’s fifth section.

Stage 2: Questionnaire design and survey. Drawing on themes identified in qualitative research and the theory of Kano model, adjustments are made to model’s structure to finalize the questionnaire. Subsequently, study participants are invited to complete the questionnaire through a combination of online (questionnaire website) and offline (paper) methods.

Stage 3: Data cleaning and analysis. Initially, collected data undergo cleaning procedures and analysis using IBM SPSS Statistics 21. Reliability and validity testing precede analysis with the traditional Kano model. Subsequently, functional quality attributes are further scrutinized by calculating the Better-Worse index and sensitivity.

### 2.1 Qualitative research

At the outset of research, a cohort of 15 older adults from China, aged 60 and above, comprising 5 males and 10 females with an average age of 67.5 years, are invited to participate in semi-structured interviews. The interviews are audio-recorded and last between 5 and 15 min each, with an average word count of 465. The interview content predominantly focuses on the following themes: (1) introducing the topic, (2) addressing basic needs and concerns, and (3) preferences of needs regarding smart home for older adults.

After the semi-structured interviews, we transcribe all audio recordings verbatim and design a categorization outline. The transcripts are manually coded in NVivo to minimize systematic coding errors, and recurring codes are compiled into codebooks. Similar codebooks are identified and categorized to distill thematic results. Finally, the results are discussed to summarize the current market situation of smart home for older adults. For example, we identify “such as children, wife of this piece of emotional communication, which is the demand of the old” as a codepoint for “Children’s Companionship” in the interview manuscript. Additionally, a code excerpt like “Emotional aspects, if there can be a simulation of children to share things with me or simulation of children and I talk or something, it is also nice that my children can communicate and talk to me through this monitor, the robot, and this kind of thing.” are identified as “Robot Chat” codepoint. Through further identification, the nodes of “Children’s Companionship” and “Robot Chat” are united under the code of “emotional needs.”

### 2.2 Questionnaire design and surveys

#### 2.2.1 Questionnaire design

To comprehensively explore older adults’ needs regarding smart home, this study utilizes a questionnaire survey methodology informed by semi-structured interviews. The questionnaire design is informed by: (1) analysis results of semi-structured interviews, (2) discussions derived from the relevant literature review. The questionnaire comprises five sections: (1) demographics; (2) health status; (3) aging styles; (4) knowledge/use of smart home for older adults; and (5) preferences for smart home for older adults features. Sections 1–4 address participants’ personal characteristics. Section 5 explores quality attribute preferences for smart home for older adults features among older adults of different age groups (60–69, 70 and above), which involves 4 themes and 15 dimensions, as outlined in Table 1.

**Table 1:** Functions and indicators for quality evaluation of smart home for older adults.

Function Number	Function	Indicator Number	Indicator
A	Life	A1	Smart security and anti-theft
		A2	Emergency contact call
		A3	Reminder to important matters
		A4	Automatic cleaning of home environment
		A5	Automatic home one-touch switch
		A6	Automatic adjustment of lights, temperature, etc.
B	Health	B1	Sleep Assistance
		B2	Remote medicine
		B3	Monitoring of body data
		B4	Customized Health Services
C	Emotion	C1	Remote accompaniment of children
		C2	Smart Voice Robot
		C3	Smart Emotional Attention
D	Entertainment	D1	Smart Home Theater/KTV
		D2	Smart personalized music/videos

According to Kano model, a total of 15 dimensions are designed into 15 pairs of forward and reverse questions (question structure: satisfaction level with the feature, satisfaction level when it is not available, and the satisfaction level, which is categorized into five options: very good, okay, indifferent, not so good, and not good). The questions in the questionnaire are presented in textual format to facilitate participants in comprehending and articulating their genuine attitudes.

#### 2.2.2 Surveys

With the population aged 60 and above expected to reach 402 million in 2040 (33), China has become one of the most representative countries facing population aging. Ministry of Industry and Information Technology of the People’s Republic of China placed significant emphasis on the development and application of smart home for older adults products (34), providing a good background for this study.

According to the World Health Organization’s definition, this study’s questionnaire targets China’s older adults aged 60 and above. Initially, a pre-survey and data testing are conducted to ensure the questionnaires met necessary criteria for formal survey. Subsequently, the formal survey is carried out from December 2022 to February 2023. Finally, a total of 454 questionnaires are collected, and 393 are deemed valid after data cleaning.

### 2.3 Kano model

The Kano model, proposed by Professor Kano of Tokyo Institute of Technology, is an effective tool for classifying and prioritizing user needs. Its key lies in analyzing the impact of user needs on user satisfaction, emphasizing the complex non-linear relationship between product performance and user satisfaction, and identifying service attributes demanded by customers (27, 35). However, the traditional Kano model can only assess customer satisfaction qualitatively, a limitation which makes it impossible to assess customer satisfaction quantitatively (36). At the same time, this data processing method has the problem that the method relies solely on the maximum frequency to determine the classification of each index attribute.

Berger et al. (37) solved the above problem by incorporating the Better-Worse index and sensitivity coefficient into the Kano model to help researchers obtain more accurate results. Therefore, this study first presents the functional attribute classification results of the traditional Kano model, and then further uses the Kano model incorporating the Better-Worse index and sensitivity coefficients to classify the attributes of smart home for older adults products features. The section 2.3.1 describes how the Kano model categorizes the attributes of the functional indicators initially, and section 2.3.2 describes the improved part of the Kano model.

#### 2.3.1 Traditional attribute classification

The Kano model transforms user satisfaction situations collected from questionnaires into quality attributes of product features. This method divides the functional properties into 5 categories: Must-be quality (M), One-dimensional quality (O), Attractive quality (A), Indifferent quality (I) and Reverse quality (R). After eliminating the Reverse quality (R), the priority order of smart home for older adults functional quality attributes is M > O > A > I (30, 32).

In this study, 15 pairs of questions are set up in the fifth section of the questionnaire, each pair of questions consisted of forward questions (equipped with function) and reverse questions (not equipped with function), for each question, respondents could choose between “very good,” “okay,” “indifferent,” “not so good,” and “not good.” There are 25 possible outcomes, each of which corresponds to an attribute, as shown in Table 2. For example, if respondent A chooses “Very good” in the forward question and “Okay” in the reverse question. The feature is classified for that respondent as “Attractive quality.”

**Table 2:** Classification table of Kano model quality attributes.

Product Features		Not equipped				
		Very good	Okay	Indifferent	Not so good	Not good
Equipped	Very good	Q	A	A	A	O
	Okay	R	I	I	I	M
	Indifferent	R	I	I	I	M
	Not so good	R	I	I	I	M
	Not good	R	R	R	R	Q

The method then counts the quality attribute classification with the highest frequency or percentage for each indicator. Subsequently, the attribute classification of a product feature is determined based on the highest frequency of its occurrence in the corresponding category.

#### 2.3.2 Attribute classification and sensitivity calculation based on the better-worse index method

To compensate for the shortcomings of the traditional Kano method, Berger et al. (37) optimized the traditional Kano model and proposed the Better-Worse index, which calculates the user’s Satisfaction Index (SI) and Dissatisfaction Index (DSI) to establish Better-Worse index coordinates. Better-Worse index elucidates the efficacy of each feature in augmenting user satisfaction and mitigating user dissatisfaction. According to the study, the categorized user needs were transformed into emotional attributes, and the degree of user need for each functional attribute was measured using satisfaction and calculated using the Better-Worse index formula. As shown in Equations 1 and 2, where A, O, M and I denote the frequency of each attribute categorization within each indicator in the questionnaire data.

Better (SI) coefficient represents the magnitude of enhancement in user satisfaction resulting from the presence of a function, with values ranging from 0 to 1. A higher SI value indicates a more substantial impact on enhancing user satisfaction. Conversely, |Worse| (|DSI|) coefficient reflects the decrease in user satisfaction when the function is absent, also ranging from 0 to 1. A higher |DSI| value suggests a weaker effect on reducing user satisfaction levels. When selecting functions for product development within the same category, the guiding principle is to prioritize those with higher SI coefficient values and lower |DSI| coefficients, signifying heightened user sensitivity towards them. S coefficient denotes the distance of a function from the origin, the greater the sensitivity the further the indicator is from the origin (30, 32). As shown in Equation 3.


(1) Better,SI=(A+O)/(A+O+M+I)

(2) Worse,DSI=−1×(O+M)/(A+O+M+I)

(3) $$S=\sqrt{\text{Bette}r^{2}+|\text{Worse}|2}$$

The functional attributes of each indicator are determined by the quadrant in which the calculated Better-Worse values fall within the Better-Worse index coordinates. Specifically, Indicators in the first quadrant are categorized as One-dimensional quality; Indicators in the second quadrant are classified as Attractive quality; Indicators in the third quadrant are defined as Indifferent quality; Indicators in the fourth quadrant are identified as must-be quality.

## 3 Results

### 3.1 Qualitative findings

After NVivo analysis, we obtain 234 reference code points, summarize them into 83 initial categories, then organize them into 14 main categories. Through analysis and comparison between the categories, we distill them into four core categories. The coding results are shown in Table 3.

**Table 3:** The codes of older adults interview transcript.

Selective coding (core categories)	Spindle code (main category)	Number of node references	Corresponding questionnaire setting section
Basic situation of older adults	Better health	15	(1) Demographics (2) Health status
	Enrichment of daily life	23	
	Problems in life	12	
Acceptance of smart home for older adults	Medium acceptance	23	(3) Aging styles (4) Knowledge/use of smart home for older adults
	Diverse factors affecting acceptance	26	
Use of smart home for older adults	Most older adults have used	15	
	Problems prevalent in use	9	
	Single type of product used	12	
	Various reasons for non-use	18	
Demand for smart home for older adults products	Basic needs	12	(5) Preferences for smart home for older adults features
	Health needs	23a	
	Emotional needs	14	
	Life needs	20	
	Entertainment needs	12	

Due to the large number of initial categories, only the core and main categories are listed in the table.

#### 3.1.1 Qualitative analysis

The qualitative analyses of the above four core categories are as follows:

Basic situations of older adults: Generally, older adults enjoy good health, but aged 70 and above are slightly weaker. Their daily lives are fulfilling and active, such as chatting, playing cards, watching TV, singing and dancing, cleaning, and physical exercise. However, some issues are reported by older adults, including memory loss, difficulty with cleaning, inability to bend down and exert themselves, and poor sleep quality.

Acceptance of smart home for older adults: Most older adults do not understand smart home for older adults, but do not outright reject it. Their acceptance is moderate, with high price being the main factor affecting their acceptance. Most older adults lack knowledge about smart home for older adults, “I do not know much about this area, these concepts are not well understood.” Some older adults have a basic understanding of the products they have used, “The products I have used are definitely still familiar to me.” For the factors affecting acceptance, older adults mainly mention price, “The price should still be moderate, because now everyone’s retirement salary and the usual consumption are not too high.” In addition, there are factors such as complexity of operation, uneven quality, and brand awareness.

Use of smart home for older adults: Some older adults have used smart home for older adults, but they often encounter common issues: “We do not remember too well now, we are afraid of forgetting passwords, which is not easy to deal with.” “Sometimes it takes a long time to learn how to operate these devices. If I do not use it for a while, I will forget, and have to ask my daughter to teach me again, which is troublesome.” Furthermore, the types of products used are limited, primarily consisting of smart TVs, monitors, door locks, watches, and other basic devices.

**Figure 2:**
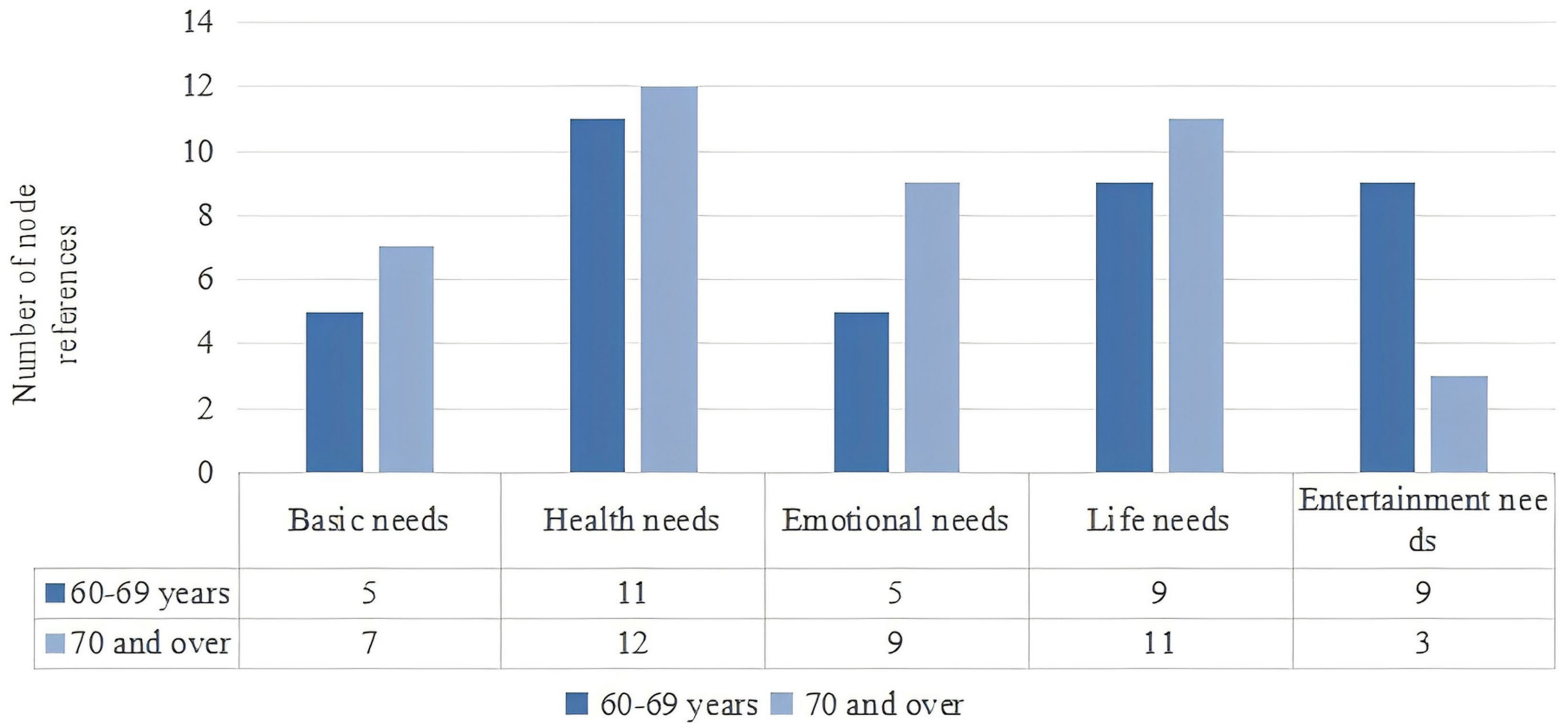
Demand for product categories by age groups.

Demand for smart home for older adults: Health functions are the most valued category for older adults, including body detection, remote medical diagnosis, and sleep monitoring. Additionally, life functions are important, including reminder systems, house cleaning, and smart security. Figure 2 shows statistics for coding points, revealing significant differences in the categories of need between two age groups of older adults. For individuals aged 60–69, there is a greater demand for entertainment because recent retirement and increased free time. For those aged 70 and above, the demand for emotional companionship is more prominent, “If the smart home can simulate having children to share my life and engage in conversation, and if children can communicate with me through monitoring or a robot, it is very good, I still crave this kind of companionship when I am old.”

#### 3.1.2 Questionnaire design supporting

The analysis results show that the number of nodes in the health category is 11 in the 60–69 age group and 12 in the 70 and above age group. Similarly, the number of nodes in the life category is 9 in the 60–69 age group and 11 in the 70 and above age group. Suggests that health and life categories are important for both age groups. However, the number of nodes in the entertainment category is 9 in the 60–69 age group and only 3 in the 70 and above age group. Additionally, the number of nodes in the emotion category is 9 in the 70 and above age group and 5 in the 60–69 age group. Both of these differences are statistically significant. When designing the questionnaire, we focus on exploring the functional needs for health, life, and entertainment for older adults aged 60–69; for those aged 70 and above, focus on the functional needs for health, life, and emotion.

The questionnaire is designed for users in different age groups, divided into two categories. Sections 1–4 of questionnaire are set up based on 1st-3rd core categories, which focus on the user’s personal characteristics. Section 5 of questionnaire is set up based on the 4th core category, which quality attribute preferences for smart home for older adults features among older adults of different age group. The combination of these sections explores the differences in product needs for different older adults.

### 3.2 Questionnaire results

In this study, Cronbach’s Alpha and KMO values of the questionnaire data are calculated by IBM SPSS Statistics 21. The results indicate that the Cronbach’s Alpha values are greater than 0.7, the KMO values are 0.924 and 0.899 for both age groups of older adults, indicating the questionnaire has a high degree of reliability.

#### 3.2.1 Frequency-based functional attribute classification results

According to subsection 3.1, the research data is divided into two stages based on age: one for the 60–69 age group and another for aged 70 and above. Life and health functions are investigated for all older adults, entertainment functions for the 60–69 age group, and emotional functions for the aged 70 and above. According to traditional attributes classification in Section 2.3.1, the quality attributes of the 12 indicators for the 60–69 age group are divided into 1 One-dimensional quality and 11 Indifferent quality. For the 70 and above age group, the quality attributes of the 13 indicators are divided into 1 One-dimensional quality, 1 Attractive quality and 11 Indifferent quality. See Table 4 for details.

**Table 4:** Quality attribute statistics for the traditional Kano model for aged 60–69 vs. 70 and above.

Age	Function Number	Function		M (%)	O (%)	A (%)	I (%)	R (%)	Q (%)	Classification
60–69	A	A1	Smart security and anti-theft	5.60%	21.60%	24.80%	46.80%	1.20%	0.00%	I
		A2	Emergency contact call	2.80%	35.20%	26.80%	35.20%	0.00%	0.00%	O/I
		A3	Reminder to important matters	2.00%	22.40%	25.60%	50.00%	0.00%	0.00%	I
		A4	Automatic cleaning of home environment	1.20%	22.80%	30.00%	45.20%	0.80%	0.00%	I
		A5	Automatic home one-touch switch	0.80%	22.00%	22.40%	54.40%	0.40%	0.00%	I
		A6	Automatic adjustment of lights, temperature, etc.	1.60%	17.20%	22.80%	58.40%	0.00%	0.00%	I
	B	B1	Sleep Assistance	0.40%	20.80%	27.60%	50.40%	0.80%	0.00%	I
		B2	Remote medicine	2.80%	22.80%	31.20%	43.20%	0.00%	0.00%	I
		B3	Monitoring of body data	0.40%	28.40%	34.40%	36.80%	0.00%	0.00%	I
		B4	Customized Health Services	3.20%	22.40%	24.40%	49.60%	0.40%	0.00%	I
	D	D1	Smart Home Theater/KTV	2.00%	11.20%	17.20%	68.80%	0.80%	0.00%	I
		D2	Smart personalized music/videos	1.60%	10.40%	24.80%	62.80%	0.40%	0.00%	I
70 and above	A	A1	Smart security and anti-theft	2.80%	22.38%	23.08%	50.35%	1.40%	0.00%	I
		A2	Emergency contact call	0.00%	40.56%	26.57%	32.17%	0.70%	0.00%	O
		A3	Reminder to important matters	2.80%	20.28%	31.47%	44.76%	0.70%	0.00%	I
		A4	Automatic cleaning of home environment	0.70%	23.78%	27.97%	46.85%	0.70%	0.00%	I
		A5	Automatic home one-touch switch	0.70%	18.88%	33.57%	45.45%	1.40%	0.00%	I
		A6	Automatic adjustment of lights, temperature, etc.	1.40%	16.08%	21.68%	60.84%	0.00%	0.00%	I
	B	B1	Sleep Assistance	0.70%	14.69%	32.17%	52.45%	0.00%	0.00%	I
		B2	Remote medicine	0.70%	27.27%	34.27%	37.06%	0.70%	0.00%	I
		B3	Monitoring of body data	0.70%	29.37%	35.66%	34.27%	0.00%	0.00%	A
		B4	Customized Health Services	2.10%	23.08%	30.07%	44.76%	0.00%	0.00%	I
	C	C1	Remote accompaniment of children	0.00%	23.78%	32.17%	42.66%	1.40%	0.00%	I
		C2	Smart Voice Robot	0.70%	14.69%	21.68%	61.54%	1.40%	0.00%	I
		C3	Smart Emotional Attention	0.70%	13.29%	20.98%	64.34%	0.70%	0.00%	I

#### 3.2.2 Functional satisfaction analysis based on better-worse and sensitivity

**Figure 3:**
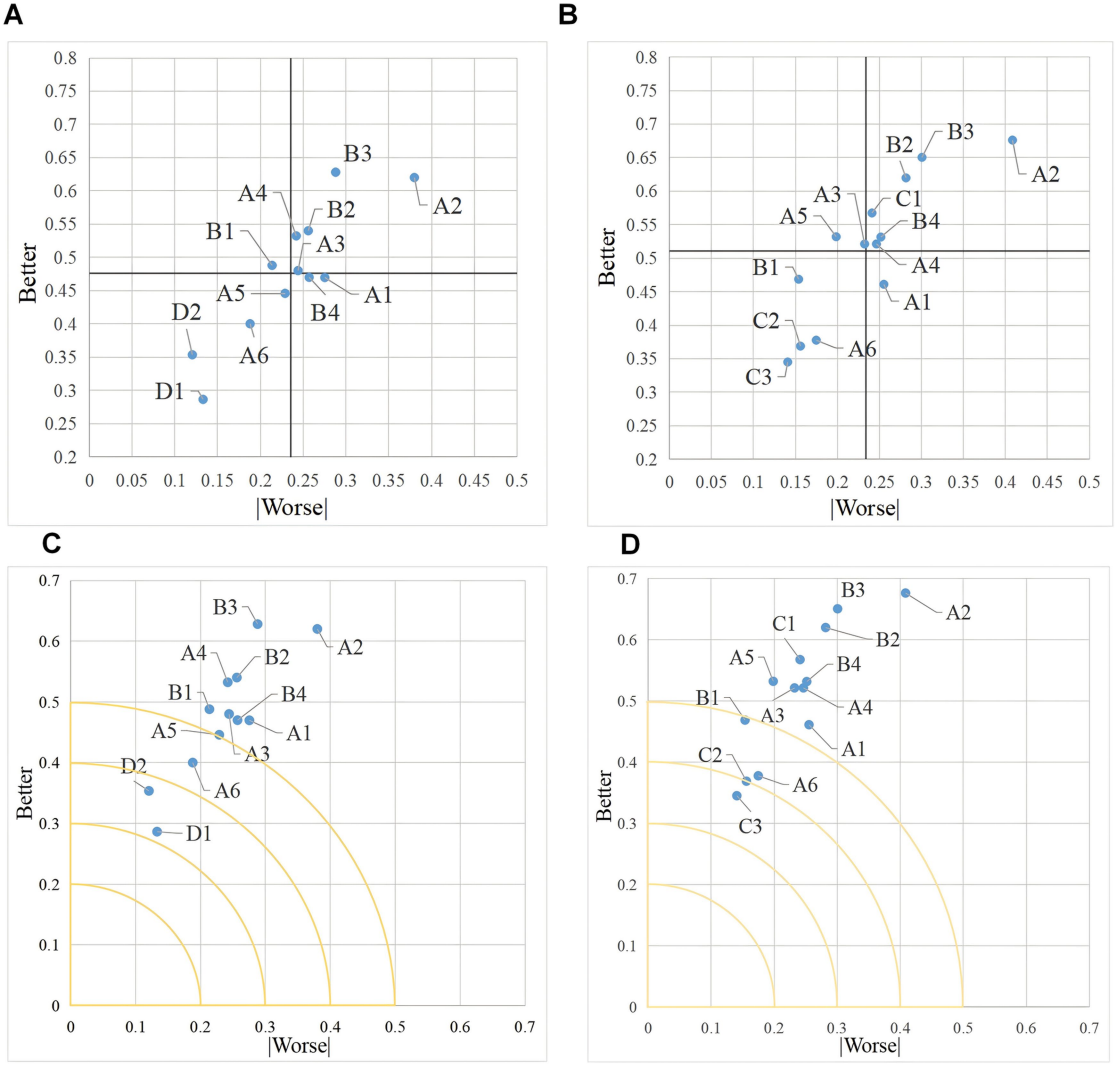
Satisfaction index statistics (60–69 vs. 70 and above). **(A)** Shows four quadrant distributions of satisfaction in 60–69 years. **(B)** Shows four quadrant distributions of satisfaction in 70 and above. **(C)** Shows sensitivity matrix chart in 60–69 years. **(D)** Shows sensitivity matrix chart in 70 and above.

Using the Better-Worse Index Method, we calculate SI and |DSI| for the two age groups respectively, and compute the functional S values using Equation 3. By averaging the SI and |DSI| values, we establish the origin point and create a four-quadrant scatter plot to display the coordinate points of each indicator (Figures 3A,B). This data processing method compensates for the traditional Kano model’s limitation that simply relies on maximum frequency to determine the quality attribute classification of each indicator. Then, we include each indicator in the sensitivity matrix, with the S value representing the distance of the function from the origin. The greater the sensitivity, the further the indicator is from the origin (Figures 3C,D).

As illustrated in Figures 3A,B, the horizontal and vertical axes represent the coordinates of the Better-Worse index. The division of each functional attribute is differentiated and categorized according to where the points in the diagram fall on the four-quadrant diagram.

The first quadrant represents One-dimensional quality, where both Better and |Worse| values are high. This indicates that a higher degree of fulfillment of these features enhances user satisfaction and prevents user dissatisfaction, and they should be prioritized for fulfillment. A2, A3, A4, B2, and B3 in the 60–69 age group, and A2, A4, B2, B3, B4, and C1 in the aged 70 and above group belong to the One-dimensional quality.

The second quadrant represents Attractive quality, with a low Better value and a high |Worse| value, indicating that such features are effective in preventing user dissatisfaction, but have no significant effect on increasing user satisfaction compared to other features. B1 in the 60–69 age group, and A3, A5 in the aged 70 and above group belong to Attractive quality. If minimizing user dissatisfaction is a priority, then such smart home for older adults product features should be refined and improved.

The third quadrant represents Indifferent quality, where both low Better and |Worse| values, indicating that such features have minimal impact on improving user satisfaction and preventing user dissatisfaction. A5, A6, D1, and D2 in the 60–69 age group, and A6, B1, C2, and C3 features in the 70 and above age group are Indifferent quality. Provision of such features may be temporarily disregarded under conditions of limited resources.

The fourth quadrant represents Must-be quality, with high Better values and low |Worse| values. These features are highly effective in improving user satisfaction but have no significant effect on eliminating user dissatisfaction. A1 and B4 in the 60–69 age group, and A1 in the aged 70 and above group are both Must-be quality. To significantly increase user satisfaction, prioritize the provision of features with higher importance.

#### 3.2.3 The analysis based on the Kano model results

Combining the evaluation results of the attributes where the functions are located, the results of Better-Worse Satisfaction Index and sensitivity matrix analysis, we summarize all the functions within each attribute sorted by priority, as shown in Table 5.

**Table 5:** Quality attribute statistics for the Kano model for aged 60–69 vs. 70 and above.

Age	Functional properties	Function Number	Product Features	Function
60–69	Must-be quality	A1	Smart security and anti-theft	life
		B4	Customized Health Services	health
	One-dimensional quality	A2	Emergency contact call	life
		B3	Monitoring of body data	health
		B2	Remote medicine	health
		A4	Automatic cleaning of home environment	life
		A3	Reminder to important matters	life
	Attractive quality	B1	Sleep Assistance	health
70 and above	Must-be quality	A1	smart security and anti-theft	life
	One-dimensional quality	A2	Emergency contact call	life
		B3	Monitoring of body data	health
		B2	Remote medicine	health
		C1	Remote accompaniment of children	emotion
		B4	Customized Health Services	health
		A4	Automatic cleaning of home environment	life
	Attractive quality	A3	Reminder to important matters	life
		A5	Automatic home one-touch switch	life

## 4 Discussion

This section discusses the findings in section 3, highlighting differences in users’ needs for smart home for older adults features based on the Kano model to provide reliable opinions for manufacturers’ design and development.

### 4.1 Research discovery

We divide the study participants into two age groups and analyze the questionnaire data using the Kano model, which helps examine the perceived degree of functionality by older adults in different age groups. Inspired by the study Liu, Y et al. (38), which explored older people’s needs for age-friendly design for a particular product, this study focuses on the needs of older people for smart home for older adults design features as a whole. The study results provide a crucial reference for manufacturers of smart home for older adults to enhance service quality.

For older adults aged 60–69, “Smart security and anti-theft” and “Customized Health Services” are Must-be quality, indicating that these services are deemed essential for smart home for older adults. This aligns with the qualitative study results, which older adults in this age group prioritizes health and life functions, particularly focusing on protecting own safety and specialized health services. They are even willing to accept more expensive products for these features. “Emergency contact call,” “Reminder to important matters,” “Automatic cleaning of home environment,” “Remote medicine,” “Monitoring of body data” are One-dimensional quality, these 5 functions cater more to emergency handling and life detail services, which are mentioned by some older adults in the interviews, but not excessively demanded. When more of these functions are provided, older adults’ satisfaction with smart home for older adults will increase. “Sleep Assistance” is Attractive quality, appealing to older adults. Although not many products on the market currently offer related services, some older adults mentioned declining sleep quality in the interviews. Meanwhile, in a study using the Kano model to explore the needs of older adults for telecare, the function of “Remote monitoring of vital signs and sleep” was a Must-be quality, indicating that older people attach more importance to the need for sleep (30).

For older adults aged 70 and above, “smart security and anti-theft” is Must-be quality, and “customized health services” is not included, indicating a shift in the needs of this age group compared to the other age group. “Emergency contact call,” “Monitoring of body data,” “Remote medicine,” “Remote accompaniment of children,” “Automatic cleaning of home environment” and “Customized health services” are One-dimensional quality. Inadequate provision of these features will diminish user satisfaction. Interview results support this finding, as most older adults aged 70 and above express a preference for filial piety, harmony among their children and health care in the interviews. The “Reminder to important matters” and “Automatic home one-touch switch” are Attractive quality, as memory loss is more pronounced in this age group, making reminder and self-help features in the life category more appealing to them.

### 4.2 Implications

The methodology and results of our paper are scientific and innovative, and have value in research. Yu et al. (9) extracted sub-functions based on a meta-analysis of existing smart home studies to obtain the smart home function preference part of the questionnaire (9). According to Liu et al. (8), the key to the design and development of smart home for older adults lies in helping older adults to establish physical and psychological connectivity with the outside world, which is subdivided into eight sub-connectivities. Further, 30 smart home subsystems and their related implementations are derived (8). Instead, based on qualitative findings and literature, we analyze the quality attribute preferences of older adults in different age groups for 15 detailed functions across 4 functional segments, revealing varying user requirements among age groups. Compared with other studies, our detailed research object division and focus on functions that users prioritize and genuinely need for satisfaction analysis. It better assists enterprises in understanding how different functions impact older adults satisfaction with smart home for older adults. This approach facilitates targeted design and development, providing valuable professional advice. Firstly, manufacturers can refine product types by designing and developing products with different functions tailored to various age groups. Prioritize quality over quantity to lower costs. Secondly, manufacturers must consider Must-be quality functions when designing and developing products. While the presence of these functions may not directly increase satisfaction, their absence can greatly diminish it. Thirdly, in the case of limited resources, the first to consider the design of One-dimensional quality of function, the higher degree of the function to improve user satisfaction, should be prioritized to be satisfied. Finally, when resources are ample, adding Attractive quality functions should be considered. Those functions can provide unexpected surprises to users, ultimately enhancing product satisfaction.

### 4.3 Limitations

First, we primarily focus on older adults of different age groups in China. Although China’s aging process and digital intelligence popularity is representative, subtle differences with other countries still cannot be ignored. Future researchers can conduct surveys on older adults in other countries in order to understand the needs related to smart home for older adults features and to improve the generalizability of study results. Secondly, we primarily base our research object categorization on the age differences among older adults. However, during qualitative research, we discovered that some older adults mentioned using smart home. Future research could classify subjects by distinguishing between those who have used smart homes and those who have not, as well as between those who are knowledgeable about smart homes and those who are not. More detailed categorization of customers will yield more scientific and unexpected conclusions.

## 5 Conclusion

In conclusion, we first identify quality evaluation functions and indicators for smart home for older adults through qualitative research and literature review. Then the Kano model is utilized to explore the classification attributes of various functions for different age groups of older adults. By classifying attributes of each product function, the attribute positioning issue in smart home for older adults functions can be addressed. This enables the development of precise product strategies tailored to the diverse needs of older users across different age groups, ultimately enhancing the life quality of older adults. To effectively implement the relevant management recommendations, companies must prioritize precise functional customization. The industry can formulate “age-function” fit combinations by leveraging the preference of different older adults age groups toward the 15 segmented functions. For instance, the 60–69 age group prioritizes health monitoring functions (e.g., customized health services, sleep assistance); Older adults aged 70 and above focus on functions such as remote accompaniment by their children and life assistance, thereby enhancing the fit of the product through precise customization. Secondly, companies must adopt dynamic resource deployment strategies, prioritizing Must-be quality features (e.g., “smart security and anti-theft” and “customized health services” for the 60–69 age group, versus “smart security and anti-theft” for those aged 70 and above). One-dimensional quality functions should be satisfied under resource constraints, with Attractive quality features addressed when resources permit.

## Data Availability

The raw data supporting the conclusions of this article will be made available by the authors, without undue reservation.
