# Supplementary material for: Exploring the functional quality attributes of smart home for older adults based on qualitative research and Kano model
Source: Front Public Health. 2025 Jul 8;13:1541571. doi: 10.3389/fpubh.2025.1541571 (PMC12279827; doi:10.3389/fpubh.2025.1541571)
Supplement: Supplementary file 1 [file Supplementary_file_1.docx]

**Interview Guide**

Dear Participant,

Hello! This study examines the preferences of older adults regarding the quality attributes of smart home for older adults’ functions. We sincerely appreciate your participation in this semi-structured interview, which will take approximately 15-20 minutes to complete. Your participation is entirely voluntary, and you are free to withdraw at any time without any repercussions. Your decision to participate or not will not lead to any negative consequences.

All information you provide will be treated with the utmost confidentiality. The data collected will be used solely for academic research purposes, and your personal information will remain strictly confidential. Should you have any questions about this study in the future, please feel free to contact us via email at [mnxlyyx@163.com](mailto:mnxlyyx@163.com) or by phone at 19980812364. By continuing with this interview, you acknowledge that you have understood the above information and consent to participate in this study.

We will sincerely appreciate your support and assistance!

**Basic Information for Interviews**

1. Interview time:

2. Place of interview:

3. Interview participants:

4. Interviewee:

5. Purpose of the interview: Elucidate the interviewee’s perspectives on smart home and their preferences regarding major smart home needs.

**Content of the interviews**

**Introduce questions (purpose: to enquire about basic conditions, e.g. health, children living with them, etc.)**

1.How has your health been recently?

2.Can you describe your daily routine?

3.Who do you currently live with?

4.How would you describe your living conditions? Are you experiencing any physical discomfort?

**Needs of older adults at different ages (food, clothing, housing, transport, recreation, health aspects, etc.)**

5. What are the specific needs you perceive at your current stage of life?

e.g. Do you find daily life monotonous? For instance, do you experience a lack of engaging recreational activities during your leisure time?

e.g. Among various needs such as nutrition, hydration, entertainment, companionship, and physical health, which do you prioritize most at this stage?

**Knowledge and Use of smart home for older adults by Older Adults**

6. Are you familiar with the concept of smart homes designed specifically for older adults, and do you have an understanding of their functionalities?

(If yes, “I have heard of/understood”)

1. Through which channels did you acquire knowledge about smart home for older adults?
2. Have you had any direct experience using smart home for older adults?

(If the answer is “used”)

1. What specific types of smart home for older adults have you utilized?
2. Which brand of smart home for older adults have you employed?
3. Could you describe your experience after utilizing the smart home for older adults?
4. Did you encounter any challenges or difficulties while using the smart home for older adults?

(If the answer is “No”)

1. What factors have influenced your decision not to utilize smart home for older adults?
2. If given the opportunity to experience a smart home for older adults, would you be inclined to try it?

(If the answer is “No”)

- What underlying reasons contribute to your reluctance to use it?

(If the answer is “I have not heard of it/do not know about it”, the interviewer will explain accordingly before continuing the question)

1. Are you open to gaining knowledge about smart home for older adults?

7. Do you have any family members or friends who have utilized smart home for older adults?

(If yes)

1. What specific type of smart home for older adults do your relatives or friends use?
2. Are you aware of the brand they have chosen?

**Older Adults’ Demand Preferences for Smart Home for older adults**

8. What features would you like to see in smart home for older adults?

a. Do you believe it is beneficial for a smart home to include features for monitoring physical health conditions? (Explore the interviewee’s preferences for health needs)

b. Do you consider it beneficial for a smart home to provide reminders for tasks you may tend to forget and offer support in managing your daily activities? (Explore the interviewee’s preferences for life needs)

c. When your children are preoccupied with work, would it be beneficial if the smart home could engage in conversations with you to alleviate feelings of boredom? (Exploring the interviewee’s preference for emotional needs)

d. Do you usually want to learn chess or other hobbies? Would it be beneficial if the smart home had the corresponding teaching function? (Explore the interviewee’s preferences for educational needs?

e. Do you think it would be beneficial for the smart home to include home cinema and karaoke functions? (Explore the interviewee’s preferences for entertainment needs)

f. In your opinion, which is more important: maintaining good health or enjoying life through good food, drink, and entertainment? (To explore the interviewee’s ranking of health needs and entertainment needs)

g. Among the functions mentioned, which do you find most appealing? Additionally, in what other ways do you hope a smart home could assist you? (Explore the major categories of needs that older adult prefer)

**INTERVIEW CONCLUSION: Thank you all for your positive co-operation!**
